# Measuring maternal mortality using a Reproductive Age Mortality Study (RAMOS)

**DOI:** 10.1186/s12884-016-1084-8

**Published:** 2016-09-29

**Authors:** Florence Mgawadere, Regine Unkels, Adetoro Adegoke, Nynke van den Broek

**Affiliations:** Centre for Maternal and Newborn Health, Liverpool School of Tropical Medicine, Pembroke Place, Liverpool, L3 5QA UK

**Keywords:** Maternal mortality ratio, RAMOS, Cause of death, Quality of care

## Abstract

**Background:**

Assessing the feasibility of conducting a prospective Reproductive Age Mortality Survey (RAMOS) study in the low-income setting of Mangochi District, Malawi to obtain cotemporaneous estimates of the number, cause of and conditions associated with maternal deaths (MD) in all women of reproductive age (WRA) (*n* = 207 688).

**Methods:**

MD among all deaths of WRA were identified using the ICD-10 definition. Cause of death and contributing conditions identified by a panel of experts using the classification system for deaths during pregnancy, childbirth and puerperium (ICD-MM).

**Results:**

Out of 424 deaths of WRA, 151 were MD giving a Maternal Mortality Ratio (MMR) of 363 per 100,000 live births (95 % CI: 307–425). Only 86 MD had been reported via existing reporting mechanisms representing an underreporting of 43 %. The majority of MD (62.3 %) occurred in a health facility and were the result of direct obstetric causes (74.8 %) with obstetric haemorrhage as the leading cause (35.8 %), followed by pregnancy-related infections (19.4 %), hypertensive disorders (16.8 %) and pregnancy with abortive outcome (13.2 %). Malaria was the most frequently identified indirect cause (9.9 %). Contributing conditions were more frequently identified when both verbal autopsy and facility-based death review had taken place and included obstructed labour (28.5 %), anaemia (12.6 %) and positive HIV status (4.0 %).

**Conclusion:**

The high number of MD that occur at health facility level, cause of death and contributing conditions reflect deficiencies in the quality of care at health facility level. A RAMOS is feasible in low- and middle-income settings and provides contemporaneous estimates of MMR.

## Background

Globally, an estimated 303,000 women die during pregnancy, childbirth and the puerperium. The vast majority (99 %) occur in low- and middle-income countries, predominantly in sub-Saharan Africa and South Asia [1]. Reducing maternal mortality (MM) is therefore one of the priority goals on the international agenda – the target for Millenium Development Goal (MDG) 5a was to reduce the Maternal Mortality Ratio (MMR) by three quarters between 1990 and 2015 and recently this target has been reset to a global target of less than 70 maternal deaths per 100,000 live births by 2030 [2, 3]. However, assessing progress towards these goals has been a challenge because less than 40 % of countries currently have complete civil registration systems with accurate attribution of cause of death necessary for reliable estimates of MMR and only two of 49 low-income countries have more than 50 % coverage of death registration [1, 3]. The new global strategy for Ending Preventable Maternal Mortality (EPMM) calls for a new approach where all maternal deaths are counted [3].

Malawi, situated in sub-Saharan Africa, is one of the fifteen sub-Saharan countries with the highest MMR (above 500 per 100,000 live births) [1]. As in most other low- and middle-income countries, there is no functioning birth and death registration system and the number of maternal deaths that occur per year is not precisely known. The available MMR estimates for such countries are based upon direct sisterhood methods as used in Demographic and Health Surveys and/or on WHO modelled estimates [1, 4]. Estimates obtained via the sisterhood method relate to the past and are indirect methods which do not identify individual deaths within a defined population. Estimates of MMR, therefore, tend to vary widely and are not contemporaneous. For example, a 2008 global review provided and estimate of MMR for Malawi of 1140 per 100,000 live-births for 2005 [5]; the Maternal Mortality Estimation Inter-Agency Group, in a report on global trends in MMR for 1990 to 2010 reported a MMR of 460 per 100,000 live births [6] while the Malawi Demographic and Health Survey (MDHS) 2010, reported a MMR of 675 per 100,000 live births [4]. Information on the causes of maternal death is even less readily available despite a new cause classification developed by WHO in 2012 [7] and efforts to scale-up Maternal Disease Surveillance and Response globally [1, 3].

The difficulties in obtaining accurate MMR estimates prompted the need to explore other methods that can provide data on MMR as well as provide information on the cause of death. A Reproductive Age Mortality Survey (RAMOS) where all deaths among women of reproductive age (WRA) are investigated is considered the best approach in the absence of vital registration data [8]. In this approach, deaths of WRA are identified using a variety of sources including health facility records, reports of community deaths and census data. Among these, all maternal deaths are identified and reviewed to identify the underlying cause of death and contributing conditions.

Our primary objective was to examine the feasibility of conducting a RAMOS in a low-income setting in sub-Saharan Africa and to see if it was possible to identify the magnitude, cause of and factors associated with MD in a defined population using this method.

## Methods

A prospective RAMOS was carried out in one entire district over one year (1st December 2011 to 30th November 2012). Mangochi District was chosen as it is considered to be one of the districts with the highest MMR in Malawi with no specific data available at the time [4]. According to the census, the district has a population of 916,274 distributed across nine traditional authorities. Of these, 207,868 were women of reproductive age (WRA). About 20 % women marry before the age of 18, the fertility rate (number of live births per 1000 population) is higher (7.0) than the national average (5.6) and literacy rates are low among women (44 %) [4, 9]. In Mangochi, the proportion of women delivering at a health facility is lower than the national average (69.3 % compared to 73 %) [4].

Maternity services are delivered at primary and secondary healthcare levels (42 health centres, 3 rural hospitals, 1 district hospital). Women can be referred to one of two tertiary hospitals in the country, the nearest of which is 110 km away. Based upon the available MMR estimates for Malawi, we expected at least 300 MD per year in the district which is considered a sufficiently large sample to be able to estimate a MMR [10].

### Estimation of the number of live births

As a civil registration system is not in place in Malawi, accurate data for number of live births for this study was unavailable, so best estimates were obtained via other sources: 1) immunization registers for BCG vaccinations 2) census report 3) using the general fertility rate obtained from the Malawi Demographic and Health Survey.

#### Estimating number of live births from immunisation registers

Empirically, the number of BCG vaccinations has been found to be a reasonable proxy for live births [11]. In Malawi, BCG vaccination is given within the first fourteen days after birth [4]. The immunisation system targets newborn babies at both health facility and community level through static clinics (at health facilities) and outreach clinics (in the community). BCG coverage at the national level is 97 % and 96 % in Mangochi District [4, 12]. Figures for each district are compiled by the Expanded Programme of Immunization (EPI) coordinators every month. Each EPI coordinator submits the figures to the Ministry of Health every quarter. For this study, the total number of BCG-vaccinated babies for Mangochi District was collected on a monthly basis from the EPI coordinator. We calculated the total monthly number of BCG vaccinations by adding all monthly reports from all 44 immunisation centres in the district. The total number of BCG-vaccinated babies documented during the study period was 39,958. This figure was increased by 1/0.96 to account for the 4 % of babies who did not receive BCG vaccine and in this way we obtained an estimated number of live births of 41,623.

#### Estimating number of live births from national census data

Census data was also used to estimate the number of live births [9]. The projected estimated total live births for Mangochi for 2012 was 43,000. This was calculated using data obtained from the National Office of Statistics [9].

#### Estimated live births using General Fertility Rate (GFR)

GFR was selected because it is considered more accurate than the crude birth rate as it represents the section of the population most likely to give birth. The GFR for rural Malawi was used as no specific data for Mangochi District was available and was estimated to be 213 per 1000 population of WRA [9]. The total number of WRA in Mangochi in 2012 as per census projection was estimated at 207,868. Therefore, using the GFR, the estimated number of births during 2012 in Mangochi was 44,276.

The RAMOS was conducted in three phases: 1) identification of deaths among WRA, 2) Identification of MD among all deaths among WRA and 3) conducting verbal autopsy and facility-based death review to obtain information on cause of death and factors associated with death.

### Identification of deaths among WRA

Identification of deaths among WRA was done after successful half-day awareness meetings at health facility and community level. At health facility level the following were oriented: The District Health Management Team, health facility in-charges, healthcare providers, zone coordinators and cluster supervisors. At community level, the Senior Health Surveillance Assistants (HSAs,) Traditional Authorities, groups village headmen and individual village headmen were oriented. Meetings were conducted over a period of three weeks at the start of the study.

The meetings were used to explain the study, agree the roles and responsibilities of each party and the set reporting system. All deaths of WRA that occurred at either facility or community level were then reported immediately after they occurred either by telephone or in writing to the research staff who were based at the district hospital.

At health facility level, deaths of WRA were identified by healthcare providers and notified to research staff if they occurred in the maternity ward or any other ward where women aged 15–49 years accessed care. In addition, all registers including the mortuary registers were reviewed. The purpose of the latter was to capture deaths which directly went to the mortuary, either after a road traffic accident or for deaths that had occurred at home and where relatives required storage of the body awaiting burial. At community level, deaths of WRA were identified via heads of households, village leaders, traditional healers, burial sites, village registers, traditional birth attendants and police stations. In addition, HSAs who are community-based healthcare workers in Malawi and expected to aggregate data on deaths that occur in the community shared information on any recorded deaths.

### Identification of MD among deaths of WRA

Upon receiving a report of any death among WRA, trained research staff obtained more information where needed using a pre-designed form to enable them to classify the death as a MD or not. Details recorded on the identification form included: woman's name, date of birth, date of death, place of death, address and the pregnancy status (which included questions as to whether the woman died during pregnancy, delivery, within 42 days after delivery/or after miscarriage or abortion). Research staff classified the death as a MD or not using the ICD-10 definition of a MD: “the death of a woman while pregnant or within 42 days of termination of pregnancy irrespective of the duration and the site of the pregnancy, from any cause related to or aggravated by the pregnancy or its management but not from accidental or incidental causes” [13]. The form also recorded the name of the data collector, name of household members who attended the last illness and death of the deceased to enable follow-up.

In order not to miss any deaths, the research staff visited all 46 healthcare facilities once a month, cross-checked all registers and checked the findings with the respective healthcare workers. At community level, quarterly review meetings were held with the HSAs to identify any deaths not yet reported. At the end of every month the lists from the two data sources, (health facilities and community), were compared and any duplicates removed. The number of MD identified in this study was compared to the number recorded in the same period via the official registration system, Health Management information System (HMIS), in the district.

### Verbal autopsy and facility-based death review

Experienced, trained research staff visited the households of all MD identified to interview all persons including Traditional Birth Attendants, neighbours, and relatives who had knowledge of the woman’s illness and death using a standard verbal autopsy questionnaire [14] which includes information on socio-economic background, place of death, treatment received for illness before death, symptoms of the deceased and the events preceding the death.

Verbal autopsy was conducted after a deliberate delay of a month in order not to intrude on the family’s period of mourning. Informed verbal consent was obtained from the deceased’s next of kin and a second respondent for each case. For women who had been treated at a health facility, information was also obtained from the patients’ case notes, the notes made at time of review of the death by the health facility audit team and through interviews with the staff who had looked after the patient. All interviews (at community and facility level) were conducted by two trained research staff who were familiar with the district and fluent in the local language.

### Assigning cause of death and contributing conditions

A panel of experts (two independent experienced obstetrician-gynaecologists and a midwife) independently analysed all information obtained via verbal autopsy and facility review (where available) to assign a single underlying cause of death and contributing conditions for each MD using the World Health Organization application of ICD-10 to deaths during pregnancy, childbirth and puerperium (ICD-MM) [7]. A classification of the cause of death was considered as satisfactory if at least two of the reviewers were in full agreement. When a different cause of death was assigned by each of the three reviewers, a panel review meeting was held. Agreement of all three reviewers was necessary in these cases to assign a final cause of death. In cases where these experts did not reach agreement, a fourth expert (Senior Obstetrician Gynaecologist with experience of working in low- and middle-income countries) was consulted.

### Statistical analysis

Statistical analysis was performed using SPSS^®^ version 21 (SPSS, Chicago, IL, USA). Fleiss’s kappa was used assess the agreement on cause of death initially assigned by each of the three expert panel members. Frequency distributions were used for categorical variables and means were used for continuous variables.

## Results

**Fig. 1:**
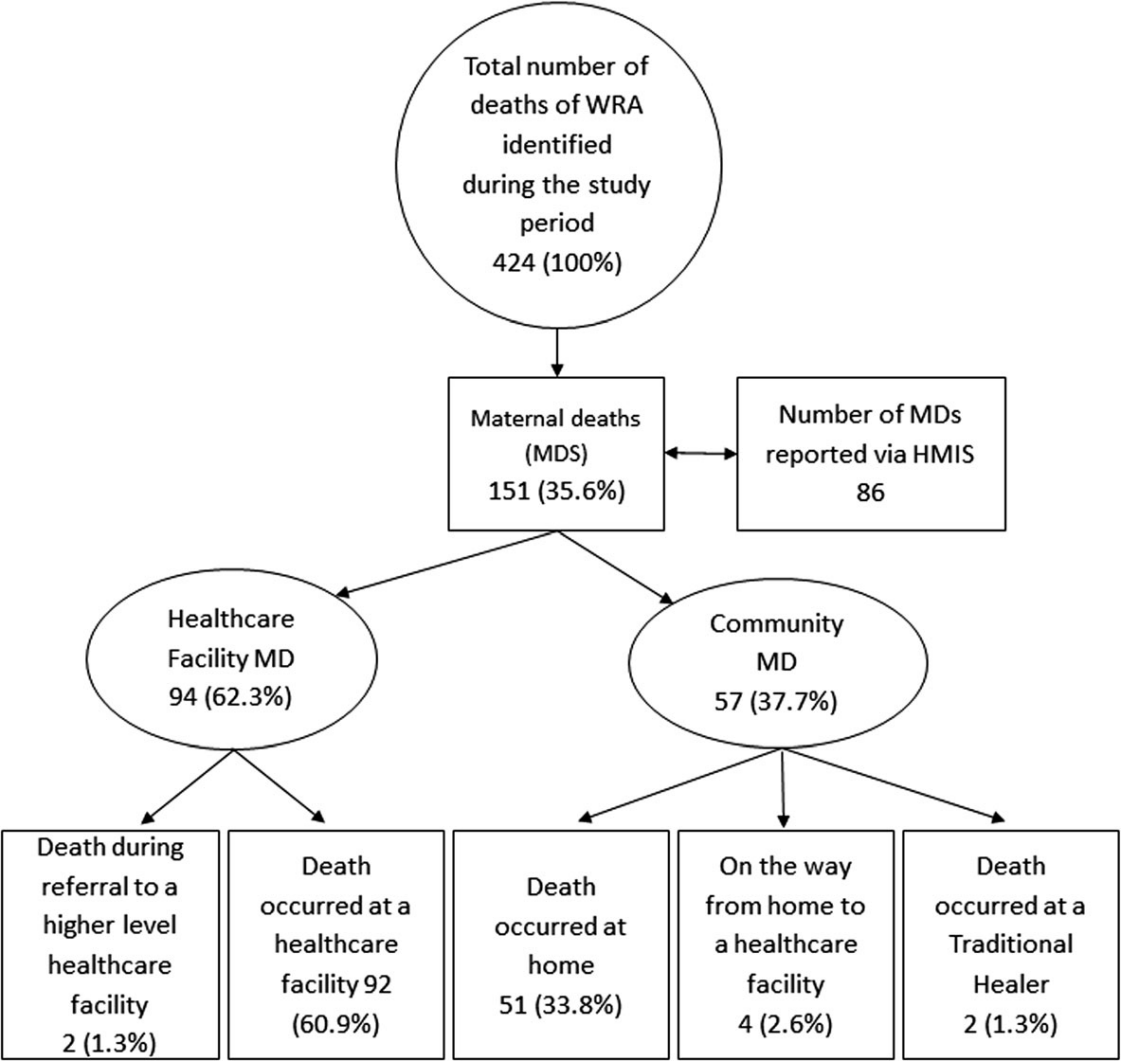
Number of deaths among women of reproductive age (WRA) and maternal deaths (MD) identified during the study period

In the 12-month period, a total of 424 women aged 15–49 years died with 151 identified to be a MD (35.6 %). Of these, 86 had been recorded via the HMIS, all of which were facility-based deaths. This study identified an additional 8 MD which had occurred at facility level which had not previously been reported and which had occurred in wards other than the maternity ward. In total, 62.3 % of MD (94/151) occurred at health facility level and 37.7 % (57/151) in the community (Fig. 1).

### Socio-demographic characteristics of maternal deaths

The mean (SD) age at the time of death was 27 (6.8) years and the age range was 15–45 years. Almost half of the deceased women did not go to school at all. The proportion of adolescent MD (15–19 years) was 15.2 % (23/151) and the number MD above 40 years 9 (6.0 %). Eighty-five percent of the women who died were married. The majority of women were multiparous (55.6 %, parity 2 to 4; and 11.9 %, parity ≥5) and 32.4 % were primiparous.

Compared to women who died at home, women who died at a health facility were younger (mean (SD) age 26 (6.5) years vs 34 (6.9) years), more likely to have attended school (79 % vs 19 %) and more likely to have their first baby (primiparity 50 % vs 3.5 %).

### Maternal mortality ratio

**Fig. 2:**
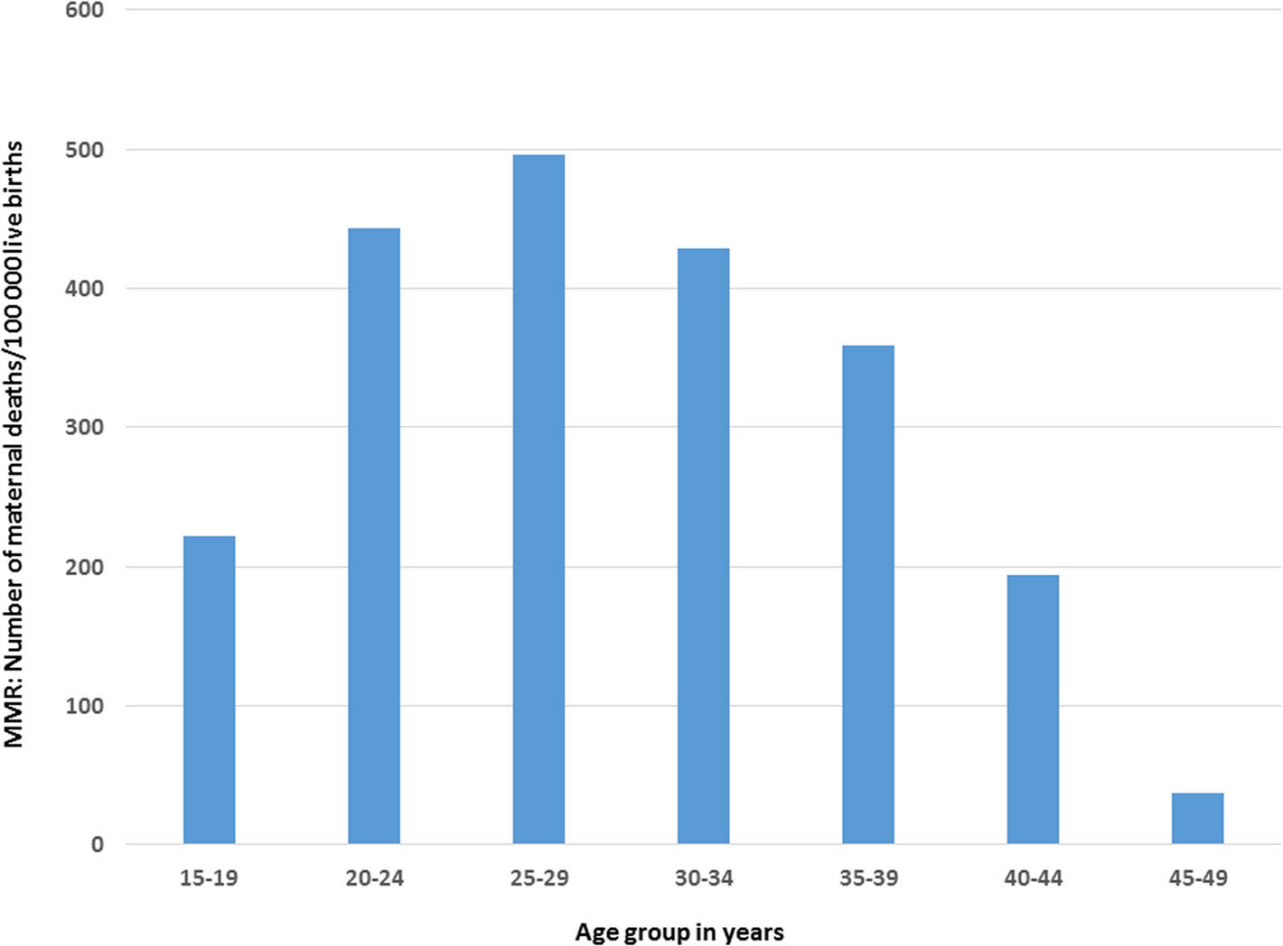
Maternal Mortality Ratio by age group

Using the most up-to-date estimate for number of births in the district (41,623 estimated using BCG coverage) the MMR was 363 deaths per 100,000 live births (95 % CI, 307–425). The MMR was highest in the age group 25–29 years (Fig. 2). Other estimates for MMR (95 % CI) using alternative estimates for number of live births are provided in Table 1.

**Table 1:** Maternal Mortality Ratio (MMR) estimates for Mangochi District obtained via a Reproductive Age Mortality Survey (RAMOS) and using different estimates of the number of live births

Source	Number of maternal deaths	Estimated number of live births	MMR: Number of maternal deaths per 100,000 live births	95 % confidence interval
Number of BCG vaccinated babies (Ministry of Health, [12])	151	41,623	363	307-425
Live births from census report, 2008 (Malawi National Statistics Office, [9])	151	43,000	351	297-412
Live births calculated from the General Fertility Rate^a^ (Malawi National Statistics Office, [4])	151	44,276	341	289-400

^a^The general fertility rate is 213 for rural Malawi (Malawi National Statistics Office, [4])

Ninety-nine women (65.6 %) died in the postpartum period, 50 (33.1 %) in the antepartum period and 2 (1.3 %), intrapartum. Among all MD which occurred in a health facility (*n* = 94), mean length of stay at the health facility was 6.5 days (SD 5.6 days). Almost three quarters of all women (73.4 % (69/94)) had been admitted for 1–7 days at a health facility before they died. About one third (27.7 % (22/94)) reached the health facility in a critical condition and of these, 31.8 % (7/22) were reported “dead on arrival”. Of the 94 deaths that occurred in a health facility, 56.4 % (53/94) were direct admissions and 43.6 % (41/94) had been referred from a health centre to a higher level of care (hospital or secondary level).

### Cause of maternal death

There was a high level of agreement for assigning a cause of death among the panel of experts. The three experts agreed on cause of death for 123/151 women (k = 0.82). Based upon the ICD-MM classification, 113 (74.8 %) of the MD were direct maternal deaths, 26 (17.2 %) were indirect MD and for 12 (7.9 %) no underlying cause of death (unclassified) could be assigned. Among direct MD, obstetric haemorrhage was the leading cause of death accounting for 47.8 % (54/113), followed by pregnancy-related infections (19.4 % (22/113)), hypertensive disorders in pregnancy, childbirth and the puerperium, (16.8 % (19/113)) and pregnancy with abortive outcome, (13.2 % (15/113)). Malaria was the leading cause in the group non-obstetric complications (Table 2).

**Table 2:** Underlying cause of death for facility-based maternal deaths as assigned by the Maternal Death Review (MDR) team, an Expert Panel (EP) and computer-based programme (InterVA-4)

ICD-MM Type	ICD-MM Group	MDR Team (%)	EP (%)	InterVA4 (%)
Direct Maternal Death	1. Pregnancy with abortive outcome	6 (7.0)	12 (14.0)	13 (15.1)
	2. Hypertensive disorders	7 (8.1)	10 (11.6)	14 (16.3)
	3. Obstetric haemorrhage	16 (18.6)	29 (33.7)	26 (30.2)
	4. Pregnancy related infections	7 (8.1)	12 (14.0)	15 (17.4)
	5. Other obstetric complications	2 (2.3)	2 (2.3)	0 (0.0)
	6. Unanticipated complications of management	1 (1.2)	1 (1.2)	1 (1.2)
Indirect Maternal Death	7. Non-obstetric complications	31 (36.0)	15 (17.4)	13 (15.1)
Unspecified	8. Unknown/undetermined	2 (2.3)	5 (5.8)	3 (3.5)
Contributing conditions		13 (15.1)	0	1 (1.2)
NC: No code available for condition in ICD-MM		1 (1.2)	0	0
Total		86 (100)	86 (100)	86 (100)

### Contributing conditions

Based upon ICD-MM, the panel of experts identified contributing conditions in 85.4 % (129/151) of all MD. This was more likely to be possible in facility-based deaths for which both verbal autopsy and case note review was possible (92/94; 97.9 %) compared to community-based deaths for whom only verbal autopsy data was available (37/57; 64.9 %).

The most common frequently identified contributing condition was obstructed labour which was noted in 28.5 % (43/151) of all MD but most frequently identified as a contributing condition during verbal autopsy for women who died in the community (17/94; 18.1 % for facility-based MD and 26/57; 45.6 % for community-based deaths). Anaemia was identified to have contributed to 12.6 % (19/151) of all MD (13/94; 13.8 % for facility-based deaths and as verified by a blood test and 6/57; 10.5 % for community-based deaths as reported via verbal autopsy and not confirmed by blood test). Among 151 maternal deaths HIV positive status was confirmed in 15 (9.9 %) cases with HIV/AIDS assigned as cause of death in six and as contributing condition in none maternal deaths. One case of tuberculosis, most likely combined with HIV positive status, was identified (Table 3).

**Table 3:** Conditions contributing to maternal death identified by a panel of experts using ICD-MM

ICD-MM code	ICD-MM name	Frequency (% of total)
Obstructed Labour		
064	Obstructed labour due to malposition and mal-presentation of the fetus	23 (17.8 %)
065	Obstructed labour due to maternal pelvic abnormality	16 (12.4 %)
066.3	Obstructed labour due to other abnormalities of the fetus (hydrocephalus)	2 (1.6 %)
064, 065	Septic shock and obstructed labour	1 (0.8 %)
075.1, 064.2	Septic shock, obstructed labour due to face presentation and prolonged labour	1 (0.8 %)
Prolonged Labour		
063.9, 075.1	Prolonged labour and septic shock	9 (7.0 %)
063	Prolonged second stage of labour	13 (10.1 %)
Anaemia		
Morbidity	Severe anaemia	15 (11.6 %)
Morbidity	Severe anaemia and heart failure	2 (1.6 %)
Morbidity, 066	Severe anaemia and obstructed labour	2 (1.6 %)
Complications of Caesarean Section		
075	Infected caesarean section wound, peritonitis	4 (3.1 %)
86.0	Infected caesarean section wound and septic shock	4 (3.1 %)
HIV		
098.7	HIV positive	6 (4.7 %)
Morbidity	Tuberculosis infection	1 (0.8 %)
Other Obstetric complications		
030	Multiple gestation	8 (6.2 %)
042	Premature rupture of membranes	7 (5.4 %)
084.4	Grand multiparity	2 (1.6 %)
062.3	Precipitate labour	2 (1.6 %)
075	Haemorrhagic/hypovolemic shock	9 (7.0 %)
7075.1	Septic shock	2 (1.6 %)
Total		129 (100 %)

## Discussion

To the best of our knowledge this is the first Reproductive Age Mortality Study (RAMOS) conducted in Malawi and we are aware of only one other RAMOS from a sub-Saharan African country to date [15]. Our findings provide the most current and comprehensive estimate of the Maternal Mortality Ratio for Malawi and demonstrate that it is feasible to conduct a RAMOS in a low-income country as it is complementary to, and strengthens existing mechanisms for maternal death surveillance and response. This study is also one of the first studies to use the ICD-MM to classify cause of and contributing factors to maternal deaths using information obtained via verbal autopsy and facility-based case reviews.

In this study, we highlight the very significant under-reporting of MD via existing reporting mechanisms. As in many other countries, existing systems currently provide unreliable data on the number and cause of MD. In Ghana, a RAMOS identified almost twice the number of MD as the officially reported number [15]. However, the number could be higher than this because the study in Ghana identified deaths of WRA (both facility-based and in the community) using hospital documentation on the assumption that all women who died at home were brought to the mortuary as this is a legal requirement for all deaths in Accra. However, there was a high possibility that some deaths were not recorded if the body was not brought to the facility. Gross underreporting exceeding 50 % was reported in India [16]. Underreporting has also been identified in high income settings including USA, Sweden, Brazil and Spain [12, 17–24]. In many cases, this is due, at least in part, to the fact that existing reporting systems only capture MD that occur in the maternity areas of a health facility.

It is well documented that MD in hospitals occur outside the maternity unit as pregnant women are admitted to other wards (e.g. women with late abortion complications to a medical ward or surgical ward) and because of direct admission for women who are pregnant or have recently given birth to wards other than the maternity ward (e.g. women with malaria or cardiac disease admitted to a medical ward) [25–29]. A RAMOS is used to systematically identify all MD among all deaths of women of reproductive age regardless of where these occurred.

We have previously demonstrated that information obtained from verbal autopsy and/or facility-based case review is usually sufficient to determine the proportion of direct and indirect maternal deaths and apply ICD-MM at least to the level of group with only 7.9 % undetermined in this study [30, 31]. Findings were in accordance with the latest global estimates with up to 85 % identified to be direct maternal deaths and haemorrhage as the commonest cause of death overall [32, 33].

Although the approach used for estimating MMR using a RAMOS in this study appears to be robust and more accurate than previous estimates, there are some limitations. We cannot be sure how many MD were missed even with the comprehensive data collection methods used and with excellent community and community health worker participation. We would contend that it is unlikely to be a frequent event except for additional MD that may have occurred in early pregnancy (complications of abortion and ectopic pregnancy) where the pregnancy had not yet been reported and/or recognised. Complications of abortion accounted for almost 10 % of all MD in this study which is slightly higher than the latest global estimate of 8 % and would suggest early pregnancy related deaths were largely identified in this study [32]. Nevertheless, in this RAMOS study and, as in other settings, such MD are likely to be underreported [34, 35]. Unsafe abortion remains a major health problem in Malawi as termination of pregnancy is only permitted in case of risk to a woman's life [36]. The most accurate denominator for calculating the MMR is the total number of all pregnancies in the population during in a given period. However, it is not possible to obtain an accurate number of all pregnancies that occurred and it is accepted practice to calculate MMR using the number of live births [37]. For this study, it was difficult to capture all live births from the registers in the district as there is no vital registration system in place to do so. The use of the proxy, numbers of babies who received a BCG vaccination, is recommended. However this was increased by 1/0.96 to account for the 4 % of babies which may not have not received BCG vaccine and/or early neonatal deaths that may have occurred before BCG vaccination could be given.

Additionally, it was not possible to conduct a verbal autopsy for all deaths of WRA and the reliance on reported signs and symptoms of pregnancy in case-notes, registers and via relatives of the deceased, means that some women with undisclosed or undiagnosed pregnancy as well as those where signs and symptoms were simply not reported/or documented could have been missed. However, the proportion of MD among women of reproductive age in our study was high (43 %) compared to that previously estimated for other countries in sub-Saharan Africa which range from 7.9 to 25.0 %). However, we note that these estimates were largely based upon modelling [1].

Women who died at a health facility were more likely to have received some schooling rather than women who died at home (79 % vs 19 %). Previous studies have shown illiteracy is a major contributor to maternal mortality [35, 38–40]. The Malawi Demographic Survey for 2010 reported a strong correlation between secondary education and skilled birth assistance (87 % compared to 60 % non-educated) [4]. Education is thought to influence health-seeking behaviour by ensuring economic empowerment, creating awareness and improved ability and freedom to make health-related decisions including choice of maternal health services during and after pregnancy and childbirth.

Findings of this study show that with increased availability of, and access to, care, the majority of MD now occur at health facility level rather than in the community. Recent studies from Nigeria, China and Bangladesh have reported that up to 82 % of MD identified occurred at health facility level [38–40].

The time during and immediately after birth is regarded as the most important or “a high risk” period. Globally, coverage with skilled birth attendance (in most cases facility-based delivery) has risen to 74 % but only 48 % of women receive postnatal care in the first two days after birth [37].

Many direct maternal deaths are preventable with timely provided emergency obstetric care which is however still not available in many low- and middle-income settings [41]. The direct obstetrical causes of death in our study, obstetric haemorrhage, sepsis and hypertensive disease with pregnancy, were similar to those of low- and middle-income countries [32, 33]. The study raises the important issue of quality emergency obstetric care available at facility level. It is important that with improved availability of, and access to care, this care is evidence-based and of good quality.

## Conclusions

To end preventable maternal deaths, the ability to count every maternal death and identify cause of death and contributing conditions is considered crucial. A RAMOS is, in our experience, an effective, feasible method that can be used to obtain such information at reasonably low cost by using the existing structures. Although in many countries a MD is now notifiable, the systems and processes in place for surveillance and review require further strengthening. This can be done alongside efforts that are underway to improve civil registration systems.

## References

[1] WHO, UNICEF, UNFPA, the World Bank and the United Nations Population Division (2015). Trends in Maternal Mortality 1990 to 2015: Estimates by the WHO, UNICEF, UNFPA, The World Bank and the United Nations Population Division.

[2] United Nations (2000). United Nations Millennium Declaration: Resolution A/RES/55/2.

[3] World Health Organization (2015). Strategies toward ending preventable maternal mortality (EPMM).

[4] Malawi National Statistical Office (2011). Malawi demographic and health survey, 2010.

[5] Hogan MC, Foreman KJ, Naghavi M (2010). Maternal mortality for 181 countries, 1980–2008: a systematic analysis of progress towards Millennium Development Goal 5. Lancet.

[6] WHO, UNICEF, UNFPA (2012). Trends in maternal mortality: 1990 to 2010: WHO, UNICEF, UNFPA and the World Bank estimates.

[7] WHO (2012). The WHO application of ICD-10 to deaths during pregnancy, childbirth and the puerperium: ICD MM.

[8] WHO, UNICEF, UNFPA (2008). Maternal mortality in 2005.

[9] Malawi National Statistical Office (2008). 2008 Population and Housing Census.

[10] WHO, UNICEF, UNFPA (2004). Maternal mortality in 2000: Estimates developed by WHO, UNICEF and UNFPA.

[11] Songane FF, Bergström S (2002). Quality of registration of maternal deaths in Mozambique: a community-based study in rural and urban areas. Social Science & Medicine Soc Sci Med.

[12] Ministry of Health (2013). 2012 Health Management Information System, Mangochi District.

[13] WHO (1992). ICD-10: International statistical classification of diseases and related health problems.

[14] UNICEF, WHO, UNFPA (1997). Guidelines for monitoring the availability and use of obstetric services.

[15] Zakariah AY, Alexander S, van Roosmalen J, Buekens P, Kwawukume EY, Frimpong P (2009). Reproductive age mortality survey (RAMOS) in Accra, Ghana. Reprod Health.

[16] Kim SY, Rochat R, Rajaratnam A (2009). Evaluating completeness of maternal mortality reporting in a rural health and social affairs unit in Vellore, India. J Biosoc Sci.

[17] Mungra A, van Bokhoven SC, Florie J, van Kanten RW, van Roosmalen J, Kanhai HH (1998). Reproductive age mortality survey to study under-reporting of maternal mortality in Surinam. Eur J Obstet Gyn R B.

[18] Kao S, Chen L-M, Shi L, Weinrich MC (1997). Underreporting and misclassification of maternal mortality in Taiwan. Acta Obstet Gyn Scan.

[19] Walraven G, Telfer M, Rowley J, Ronsmans C (2000). Maternal mortality in rural Gambia: levels, causes and contributing factors. Bull World Health Organ.

[20] Fortney JA, Susanti I, Gadalla S, Saleh S, Rogers SM, Potts M (1986). Reproductive mortality in two developing countries. Am J Public Health.

[21] Olsen BE, Hinderaker SG, Lie RT, Bergsjø P, Gasheka P, Kvåle G (2002). Maternal mortality in northern rural Tanzania: assessing the completeness of various information sources. Acta Obstet Gyn Scan.

[22] Alves SV (2007). Maternal Mortality in Pernambuco, Brazil: What Has Changed in Ten Years?. Reprod Health Matter.

[23] Esscher A, Högberg U, Haglund B, Essën B (2013). Maternal mortality in Sweden 1988–2007: more deaths than officially reported. Acta Obstet Gyn Scan.

[24] Deneux-Tharaux C, Berg C, Bouvier-Colle MH, Gissler M, Harper M, Nannini A (2005). Underreporting of pregnancy-related mortality in the United States and Europe. Obstet Gynecol.

[25] Dao B, Rouamba A, Ouédraogo D, Kambou T, Bazié AJ (2003). Transfert de patientes en état gravido-puerpéral en réanimation: à propos de 82 cas au Burkina Faso [Transfer of obstetric patients in a pregnant and postpartum condition to an intensive care unit: eighty-two case in Burkino Faso]. Gynecol Obstet Fertil.

[26] Mbaruku G, Bergström S (1995). Reducing maternal mortality in Kigoma, Tanzania. Health Policy Plann.

[27] Goswami D, Rathore AM, Batra S, Dubey C, Tyagi S, Wadhwa L (2013). Facility-based review of 296 maternal deaths at a tertiary centre in India: Could they be prevented?. J Obstet Gynaecol Re.

[28] Qomariyah SN, Pambudi ES, Anggondowati T, Latief K, Achadi EL, Bell JS (2009). A practical approach to identifying maternal deaths missed from routine hospital reports: Lessons from Indonesia. Glob Health Action.

[29] Sombie I, Meda N, Hounton S, Bambara M, Ouedraogo TW, Graham W (2007). Missing maternal deaths: lessons from Souro Sanou University Hospital in Bobo-Dioulasso, Burkina Faso. Trop Doct.

[30] Ameh CA, Adegoke A, Pattinson R, van den Broek M (2014). Using the new ICD-MM classification system for attribution of cause of maternal death-a pilot study. BJOG.

[31] Owolabi H, Ameh C, Bar-Zeev S, Adaji S, Kachale F, van den Broek N (2014). Establishing cause of maternal death in Malawi via facility-based review and application of the ICD-MM classification. BJOG.

[32] Say L, Chou D, Gemmill A, Tunçalp Ö, Moller AB, Daniels J (2014). Global causes of maternal death: a WHO systematic analysis. Lancet Glob Health.

[33] Kassebaum NJ, Bertozzi-Villa A, Coggeshall MS, Shackelford KA, Steiner C, Heuton KR (2014). Global, regional, and national levels and causes of maternal mortality during 1990–2013: a systematic analysis for the Global Burden of Disease Study 2013. Lancet.

[34] Horon IL (2005). Underreporting of Maternal Deaths on Death Certificates and the Magnitude of the Problem of Maternal Mortality. Am J Public Health.

[35] Gerdts C, Vohra D, Ahern J, Baradaran HR (2013). Measuring Unsafe Abortion-Related Mortality: A Systematic Review of the Existing Methods. PLoS One.

[36] Government of Malawi (1930). Law of Malawi: Malawian Penal Code Chapter 7:01.

[37] World Health Organization (2015). World Health Statistics 2015.

[38] Adegoke AA, Campbell M, Ogundeji MO, Lawoyin T, Thomson AM (2013). Place of birth or place of death: an evaluation of 1139 maternal deaths in Nigeria. Midwifery.

[39] Yang S, Zhang B, Zhao J, Wang J, Flick L, Qian Z (2014). Progress on the Maternal Mortality Ratio Reduction in Wuhan, China in 2001–2012. PLoS One.

[40] Halim A, Utz B, Biswas A, Rahman F, van den Broek N (2014). Cause of and contributing factors to maternal deaths; a cross-sectional study using verbal autopsy in four districts in Bangladesh. BJOG.

[41] Ameh CA, Msuya S, Hofman J, Raven J, Mathai M, van den Broek N (2012). Status of Emergency Obstetric Care in Six Developing Countries Five Years before the MDG Targets for Maternal and Newborn Health. PLoS One.

